# Stress-Induced Executive Dysfunction in GDNF-Deficient Mice, A Mouse Model of Parkinsonism

**DOI:** 10.3389/fnbeh.2016.00114

**Published:** 2016-06-21

**Authors:** Mona Buhusi, Kaitlin Olsen, Benjamin Z. Yang, Catalin V. Buhusi

**Affiliations:** Department of Psychology, Interdisciplinary Program in Neuroscience, Utah State UniversityLogan, UT, USA

**Keywords:** chronic unpredictable stress, decision making, glial-derived neurotrophic factor, impulsivity, nucleus accumbens, orbitofrontal cortex, Parkinson’s Disease, temporal discounting

## Abstract

Maladaptive reactivity to stress is linked to improper decision making, impulsivity, and discounting of delayed rewards. Chronic unpredictable stress (CUS) alters dopaminergic function, re-shapes dopaminergic circuits in key areas involved in decision making, and impairs prefrontal-cortex dependent response inhibition and working memory. Glial-derived neurotrophic factor (GDNF) is essential for regulating dopamine (DA) release in the basal ganglia and for the survival of dopaminergic neurons; GDNF-deficient mice are considered an animal model for aging-related Parkinsonism. Recently, GDNF expression in the striatum has been linked to resilience to stress. Here we investigated the effects of CUS on decision making in GDNF-heterozygous (HET) mice and their wild-type littermate controls (WT). Before CUS no differences in temporal discounting (TD) were found between genotypes. However, following CUS GDNF HET mice, having a partial reduction of GDNF levels, showed increased impulsive choice indexed by a reduction in percent Larger-Later (LL) choices in the TD paradigm, and a reduction in area under the TD curve. Moreover, stressed GDNF HET mice, but not their WT controls, showed decreased neuronal activation (number of cFos positive neurons) in the orbitofrontal cortex (OFC), nucleus accumbens (NA) core, and NA shell, suggestive of a maladaptive response to stress. Interestingly, area under the TD curve positively correlated with cFos activation in the NA core, and NA shell, but not with orbitofrontal activity. These results provide further evidence of the differential involvement of the OFC, NA core, and NA shell in impulsive choice, and identify GDNF-deficient mice as a double-hit (gene × environment) model of stress-related executive dysfunction, particularly relevant to substance abuse and Parkinson’s disease (PD).

## Introduction

Stress—defined as a real or perceived threat to homeostasis or well-being of the organism—initiates adaptive processes to promote survival. Activation of multiple interacting processes, from biochemical, endocrine and immune responses to behavioral changes, produces an integrated stress response. While initially adaptive, persistent or pronounced molecular changes engaged by these systems can have long-term deleterious implications for health and survival. Maladaptive reactivity to stress is linked to improper decision making, impulsivity, and discounting of delayed consequences (Dias-Ferreira et al., 2009; George and Koob, 2010; Jezierski et al., 2014; Wang et al., 2014), leading to substance abuse (Decressac et al., 2011; Miller, 2011; Ortiz-Ortiz et al., 2011) and abnormal behavior (Starcke and Brand, 2012; Littrell et al., 2013). In humans, acute exposure to psychosocial stress (Bickford et al., 2001) and increased levels of biomarkers of stress are associated with increased rates of discounting (Fields et al., 2009; Diller et al., 2011), and are predictive of vulnerability to substance abuse (Airavaara et al., 2004; Shevtsova et al., 2006; Dennhardt and Murphy, 2011). In contrast, exposure to stress-relieving stimuli, like natural scenes (Hauck et al., 2006), decreases impulsive decision making in the *temporal discounting* (TD) paradigm in human participants. In rodents, chronic exposure to stress hormones during adolescence, increases impulsivity in a TD task (Hubbard et al., 2009). *Chronic unpredictable stress* (CUS) alters *dopamine* (DA) release and metabolism (Ahmad et al., 2010), re-shapes fronto-striatal circuitry involved in decision making (Dias-Ferreira et al., 2009), and impairs prefrontal cortex-dependent response inhibition and working memory (Revilla et al., 2014).

*Glial-derived neurotrophic factor* (GDNF) is a member of the Transforming Growth Factor-beta superfamily of neurotrophic factors, with particular importance for dopaminergic neurons. GDNF is required for dopaminergic neuron survival, expression of enzymes required for DA synthesis (such as tyrosine hydroxylase), and high affinity DA uptake (Lin et al., 1993). Several lines of genetically engineered mice have been developed to better explore the role of GDNF and its receptors in dopaminergic neuron development and survival (Tomac et al., 1995; Pichel et al., 1996; Kramer et al., 2007; Pascual et al., 2008). Total GDNF knockout leads to immediate postnatal death due to kidney agenesis (Pichel et al., 1996). A partial reduction of GDNF levels in GDNF *heterozygous* (HET) mice (65% of GDNF levels seen in age matched wild-type (WT) littermates) leads to an accelerated aging-related decline of DA and motor function (Boger et al., 2006; Griffin et al., 2006).

*Nucleus accumbens* (NA)-derived GDNF is a retrograde enhancer of dopaminergic tone in the mesocorticolimbic system (Wang et al., 2010). GDNF expression is increased in the mouse hippocampus during CUS as well as during recovery (Bian et al., 2012). Uchida et al. (2011) found that epigenetic regulation of GDNF expression in the NA influences vulnerability to CUS: individuals who cannot upregulate GDNF during stress exhibit anxiety, anhedonia and avoidance of social interactions, possibly due to the negative consequences of chronic stress on the dopaminergic circuits.

Therefore, we hypothesized that following CUS, *GDNF-deficient* HET mice would be less able to increase levels of GDNF (due to having a single functional allele) than their WT littermates, with negative consequences on dopaminergic function and decision making. Here we investigated decision making in GDNF HET male mice and their WT littermate controls before and after exposure to CUS in the TD paradigm. In order to evaluate functional alterations in corticolimbic circuits in stressed GDNF mice, we also analyzed neuronal activation (measured by cFos expression) in the NA, the *orbitofrontal cortex* (OFC), and the *prelimbic cortex* (PrL), and their correlation with impulsive choice.

## Materials and Methods

### Subjects

The subjects were 27 6–8 months-old male GDNF-deficient (HET, *n* = 12) mice and their WT (*n* = 15) littermate controls from a GDNF colony (Granholm et al., 1997) maintained on C57BL/6 background for at least 10 generations. Genotypes were confirmed by PCR amplification from tail biopsy samples. Mice were housed in a temperature-controlled room under a 12-h light-dark cycle. Mice were maintained at 85% of their *ad libitum* weights by restricting access to food (Teklad Diet 8064, Harlan Laboratories Inc., Indianapolis, IN, USA); weight did not differ between groups either before or after stress manipulations (*t*s_(25)_ < 0.44, *p* > 0.05). All manipulations were performed in compliance with ethical standards for the treatment of animals National Research Council [USA] (2011) and were approved by Utah State University IACUC committee.

### Apparatus

The behavioral setup consisted of 12 mouse operant chambers (Med Associates, St. Albans VT, USA) equipped with a food cup and a white noise generator/speaker on the front wall, and two nosepokes, a lever (between the nosepokes), and a house light (above the lever) on the opposing wall. Noyes precision food pellets 20 mg (Research Diets, Inc., New Brunswick, NJ, USA) were delivered in the food cup according to the paradigm.

### Procedures

#### Pre-stress TD Paradigm

After being shaped to nose-poke and lever-press for food pellets, mice were trained in a TD paradigm modified after Adriani and Laviola (2003) and Isles et al. (2003). Briefly, mice were presented with two alternatives, Smaller-Sooner (SS), 1 pellet at 0 s delay, and Larger-Later (LL), four pellets at progressively larger delays. Sessions consisted of 40 trials broken up into five 8-trial blocks. The beginning of a block was signaled by the house light flashing for 1 min; continuous illumination of the house light signaled that the mice can self-initiate a trial by pressing on the lever. Each block consisted of six forced choice trials (3 pairs of forced-choice trials on the SS and LL alternatives), followed by two free-choice trials between alternatives. During forced-choice trials, upon lever pressing, one nosepoke was lit and the subject had to respond on that nosepoke to receive the appropriate reward. For free-choice trials both nosepokes were lit and the subject was free to choose either nosepoke to receive the associated reward. Upon choosing the nosepoke, the nosepoke flashed during the delay period between choice and reward delivery (cued delay). If mice failed to initiate a trial within 30 s after the house light was turned on continuously, or if no nosepoke was recorded within 30 s of nosepoke illumination, the trial was terminated by a 2-s blackout (inter-trial interval). The position of the SS and LL nosepokes (to the left or to the right of the lever) was counterbalanced among subjects. For each session, the five blocks of trials differed by the delay on the LL choice, presented in increasing order of delay during each session. Mice received five sessions with 0 s LL delays, five sessions with the LL delays 0 s, 1 s, 2 s, 4 s, 8 s, and five sessions with the LL delays 0 s, 4 s, 8 s, 16 s, 32 s. Mice were then tested during four sessions with the LL delays 0 s, 4 s, 16 s, 64 s. Data from these four pre-stress test TD sessions were subjected to data and statistical analyses.

#### Chronic Unpredictable Stress (CUS)

After being tested in the TD paradigm, all mice were subjected to a CUS paradigm for 21 days as in Dias-Ferreira et al. (2009). Briefly, mice were exposed once daily to one of the following stressors (randomly chosen): 30 min restraint in a small container, 10 min forced swim, or 10 min exposure to an aggressive BALB/cJ male mouse (Brodkin, 2007).

#### Post-Stress TD

After CUS, mice were re-tested for four sessions in the TD paradigm with the LL delays 0 s, 4 s, 16 s, 64 s, as described above. Data from these four post-stress TD sessions were subjected to data and statistical analyses.

#### cFos Immunostaining

To assess neuronal activation, 2 h after the start of the last TD test session mice were deeply anesthetized with isoflurane and transcardially perfused with a paraformaldehyde solution (4% in 0.1 M phosphate buffer, pH 7.4). Brains were collected and sectioned on a vibrating microtome (VT1200S, Leica, Germany). cFos immunostaining was performed using standard procedures similar to Bertran-Gonzalez et al. (2008). Free-floating brain sections (50 μm) were incubated with a blocking and permeabilization solution (10% donkey serum, 0.3% Triton X-100 in PBS) for 2 h and then incubated overnight at 4°C with the cFos primary antibody (Cell Signaling Technologies, 1:300 dilution). Sections were rinsed in PBS, 0.1% Tween-20 and incubated for 2 h with Alexa 488 conjugated donkey anti rabbit secondary antibody and Neurotrace 530/615 (Life Technologies). Neurotrace neuronal labeling was used to identify the neuroanatomical regions of interest. Sections were rinsed in PBS before mounting with Prolong Gold (Life Technologies).

### Data Analysis

#### Behavioral Data Analysis

Behavioral data was collected using Med-PC software (Med Associates, St. Albans VT, USA). The percent of LL options chosen by the subjects in the free-choice trials at each delay was averaged over sessions and subjected to statistical analyses. The TD curve was also normalized both in the y (%LL) and x (delay) axes as in Myerson et al. (2001), and the percent area under the normalized TD curve (%AUC), a global measure of impulsivity at all delays (Myerson et al., 2001), was also computed and submitted to statistical analyses: the smaller the %AUC, the steeper the discounting, and the more impulsive the individual.

#### Neural Activation Analysis

Image acquisition and neuronal activation analysis was performed on a Zeiss LSM710 laser scanning confocal microscope. Double-labeled images from the regions of interest—OFC, PrL, NA core and NA shell—were obtained using appropriate filter sets. Analysis of neuronal activation was performed by counting cFos-positive nuclei, in corresponding areas in two sections/region of interest/mouse (bregma 2.22/2.68 for OFC, bregma 1.94/2.34 for PrL, bregma 1.34/1.78 for NA core, and bregma 1.10/1.42 for NA shell; Franklin and Paxinos, 2008), by two independent observers unaware of genotype; Pearson’s *r* correlation (inter-reliability) between observers was *r* = 0.32, *p* < 0.01. Neuronal activation in each region was averaged over observers and subjected to statistical analyses.

#### Statistical Analyses

The %LL choices were submitted to mixed ANOVAs with between-subjects variable genotype (HET, WT) and within-subject variables stress (pre and post) and delay (0 s, 4 s, 16 s, 64 s), followed by planned and *post hoc* analyses. The %AUC was subjected to mixed ANOVAs with between-subjects variable genotype (HET, WT) and within-subject variables stress (pre and post), followed by planned and *post hoc* analyses. The individual average neuronal activation (cFos+ counts) for each region of interest was submitted to *t*-tests with between-subjects variable genotype (HET, WT). Pearson’s* r* correlation coefficient was estimated between %AUC and neuronal activation (cFos+ counts) in each region of interest. Analyses were conducted in STATISTICA 6.0 (StatSoft, Tulsa OK, USA), with a 0.05 alpha level.

## Results

### Unpredictable Stress Increases Impulsivity in GDNF HET Mice Relative to WT Controls

**Figure 1:**
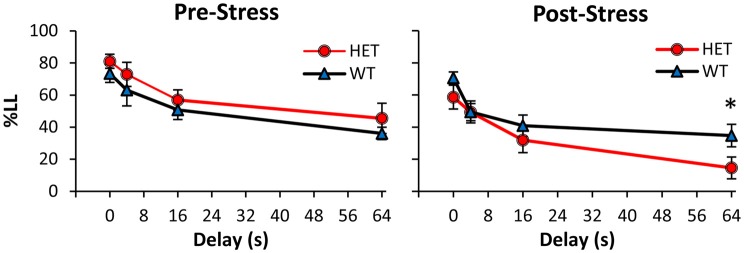
**Increased temporal discounting (TD) in GDNF-deficient mice after chronic stress.** Average % larger-later (LL) choices (± SEM) in GDNF-deficient heterozygous (HET, *n* = 12) and wild-type (WT) controls (*n* = 15) before (left) and after chronic unpredictable stress (CUS; right). *p < 0.05.

Mice were tested in the TD paradigm before stress (Pre-Stress condition, Figure 1 left panel), after which they were exposed for 21 days to a CUS stress paradigm and then re-tested in the TD paradigm (Post-Stress condition, Figure 1 right panel). Before CUS, analyses of %LL choices indicated a main effect of delay (*F*_(3,75)_ = 43.10, *p* < 0.01), suggesting that all mice acquired the TD task and discounted in a delay-dependent fashion, with no discounting differences between genotypes (all *F*s_(1,25)_ < 1.21, *p* > 0.05). However, following exposure to stress (Figure 1 right panel), GDNF HET mice made fewer LL choices at the longest 64 s delay (*F*_(1,25)_ = 5.51, *p* < 0.05), but not at shorter delays (all *F*s_(1,25)_ < 3.3, *p* > 0.05). Taken together, these analyses failed to identify discounting differences between genotypes before stress, but suggest that after stress GDNF-HET mice discounted more than WT controls.

Analyses of %LL choices further indicated a main effect of stress (*F*_(1,25)_ = 18.37, *p* < 0.01), suggesting that the CUS regimen resulted in overall fewer LL choices in the Post-Stress condition relative to the Pre-Stress condition, though this effect was mainly seen in the GDNF HET mice. This suggestion was supported by a reliable stress × genotype interaction (*F*_(1,25)_ = 5.97, *p* < 0.05). Planned comparisons (Pre-Stress, Figure 1 left panel vs. Post-Stress, Figure 1 right panel) supported the suggestion that the two genotypes were differentially affected by stress. Following exposure to stress, GDNF HET mice discounted reliably more than before stress at all delays (all *F*s_(1,25)_ > 6.43, *p* < 0.05), while WT controls were unaffected by stress (all *F*s_(1,25)_ < 0.85, *p* > 0.05), suggesting that GDNF-deficient mice, but not their WT controls, were sensitive to the effect of chronic stress.

**Figure 2:**
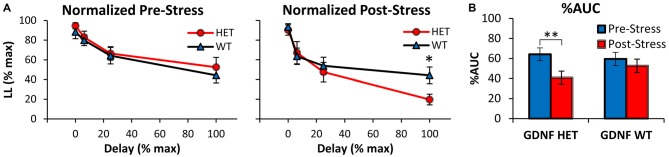
**Decreased %AUC in GDNF-deficient mice after chronic stress. (A)** Average normalized %LL choices (± SEM) in GDNF-deficient (HET, *n* = 12) and WT controls (*n* = 15) before (left) and after CUS (right). **(B)** Average %AUC (± SEM) in GDNF-deficient mice (HET) and WT controls in the Pre- and Post-Stress conditions. *p < 0.05, **p < 0.01.

Similar conclusions were reached after normalizing the discounting curves both in the x (delay)- and y (%LL)- axes as shown in Figure 2A, in order to compute and analyze the %AUC (Myerson et al., 2001). Indeed, analyses of the normalized discounting curves (Figure 2A) confirmed the main effect of delay (*F*_(3,75)_ = 43.79, *p* < 0.01), suggesting that mice acquired the TD task and discounted in a delay-dependent fashion. Before stress (Figure 2A left panel) no normalized discounting differences were found between genotypes (all *F*s_(1,25)_ < 0.56, *p* > 0.05). However, following exposure to stress (Figure 2A right panel), GDNF HET mice made fewer LL choices at the maximal delay (100% normalized delay, 64 s; *F*_(1,25)_ = 5.28, *p* < 0.05), but not at shorter delays (all *F*s_(1,25)_ < 0.26, *p* > 0.05). Taken together, these analyses failed to find discounting differences between genotypes before stress, but suggest that after stress GDNF HET mice discounted at a higher rate than WT controls.

Figure 2B shows the %AUC in GDNF HET and WT mice in the Pre- and Post-Stress conditions. Analyses indicated a main effect of stress (*F*_(1,25)_ = 8.16, *p* < 0.01), although the effect of stress seemed to be prominent in the GDNF HET mice but not in the WT controls (Figure 2B). Indeed, %AUC decreased reliably Post-Stress in HET mice (*F*_(1,25)_ = 8.81, *p* < 0.01), but not in WT controls (*F*_(1,25)_ = 0.93, *p* > 0.05). Taken together, these results suggest an increased vulnerability to stress (reduced %LL choices, increased discounting rate, reduced %AUC, and increased impulsivity) in GDNF HET mice, but not in their WT littermate controls.

### Decreased Post-Stress Neuronal Activation in GDNF HET Mice Relative to WT in the OFC, NA Core, and NA Shell

**Figure 3:**
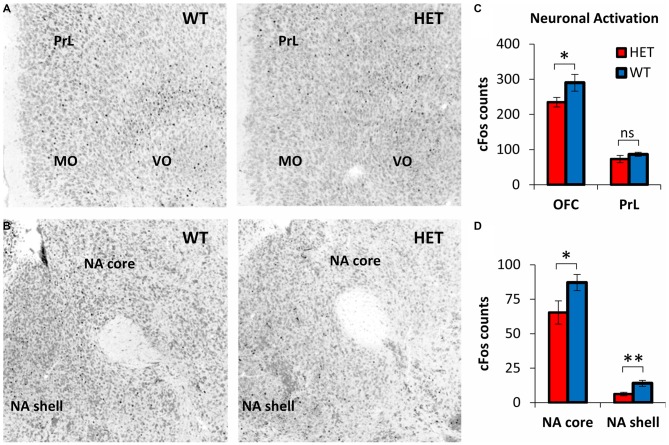
**Decreased neuronal activity during post-stress TD in GDNF-deficient mice relative to controls. (A)** Representative orbitofrontal cortex (OFC) and prelimbic cortex (PrL) cFos expression in GDNF-deficient mice (HET) and WT controls in the Post-Stress condition. Images were converted to grayscale for better contrast. The Neurotrace stain used to identify neurons and neuroanatomical regions is shown in gray; cFos immunostaining appears as black dots. **(B)** Representative nucleus accumbens (NA) core and NA shell cFos expression in GDNF HET and WT controls in the Post-Stress condition (see **A** for details). **(C)** OFC and PrL neuronal activity (average cFos+ cell counts ± SEM) in GDNF HET (*n* = 7) and WT controls (*n* = 7) in the Post-Stress condition. **(D)** NA core and NA shell neuronal activity (average cFos+ cell counts ± SEM) in GDNF HET (*n* = 7) and WT controls (*n* = 7) in the Post-Stress condition. ns *p* > 0.05; *p < 0.05; **p < 0.01.

Neuronal activation during TD was evaluated by cFos+ cell counts in OFC, PrL, NA core, and NA shell, brain regions with relevant roles in decision making (da Costa Araujo et al., 2010). Figure 3A shows representative cFos immunostaining in OFC and PrL, and Figure 3B shows representative cFos immunostaining in NA core and NA shell during TD in the Post-Stress condition, in GDNF HET mice (right) and WT controls (left). For better contrast, images were converted to grayscale: the Neurotrace stain used to identify neurons and neuroanatomical regions is shown in gray; cFos immunostaining appears as black dots. Figures 3A,B indicate a reduced neuronal activation in OFC, NA core and NA shell in GDNF HET mice (right) relative to their WT controls (left).

This suggestion was supported by statistical analyses of average cFos+ neuronal counts in GDNF HET (*n* = 7) and WT controls (*n* = 7) in OFC and PrL (Figure 3C) and in NA core and shell (Figure 3D): neuronal activation (cFos+ cell counts) decreased reliably in the Post-Stress condition in GDNF HETs relative to WT controls in OFC (*t*_(12)_ = 2.98, *p* < 0.05), NA core (*t*_(12)_ = 3.08, *p* < 0.05), and NA shell (*t*_(12)_ = 4.56, *p* < 0.01), but not in PrL (*t*_(12)_ = 1.66, *p* > 0.05; Figure 3C). These results suggest that the decrease in neuronal activation in GDNF HET mice is specific to regions previously shown to be involved in the TD task, rather than being a general brain-wide effect.

### Area Under the TD Curve Positively Correlated with Neural Activation in NA Core and NA Shell, but not in OFC or PrL

Pearson’s* r* correlation coefficient between %AUC and neuronal activation (cFos+ cell counts) in OFC, PrL, NA core and NA shell was estimated in GDNF mice over both genotypes (*n* = 14) in the Post-Stress condition. Analyses indicated that %AUC positively correlated with neural activation in NA core (*r*_(12)_ = 0.57, *p* < 0.05) and NA shell (*r*_(12)_ = 0.68, *p* < 0.01), while no correlation was observed with orbitofrontal (*r*_(12)_ = 0.05, *p* > 0.05) or prelimbic activity (*r*_(12)_ = 0.03, *p* > 0.05). These results suggest that in our TD task Post-stress impulsivity (reduced %AUC) negatively correlated with neuronal activation in the accumbens, but not in OFC or PrL.

## Discussion

The current study evaluated the effects of stress on decision making in mice using a cued-delay solid-food reward TD paradigm. Most procedures currently used to assess TD in mice are based on an adjusting alternative procedure (Richards et al., 1997), and use liquid rewards (Mitchell, 2014), in which subjects indicate their preference for an “adjusting” alternative relative to the “standard” alternative. In these procedures, an indifference point is calculated, and a TD function is generated, describing how changes in delay affect the subjective value of the standard reward. Another type of procedure, widely used with rats but rarely with mice, is the within-session procedure (Evenden and Ryan, 1996; Isles et al., 2003), in which the main independent variable is the number of trials where the LL alternative is chosen over the SS alternative within a block of trials. The LL delay increases systematically across trial blocks, permitting TD to be estimated via shifts in preference indicated by changes in the frequency of delayed alternative choices across blocks. Adriani and Laviola (2003) developed a procedure in which food-restricted mice were tested in operant chambers with two nosepokes that delivered one food pellet immediately (SS) or five pellets after a delay (LL), respectively. We have modified the procedures of Adriani and Laviola (2003) and Isles et al. (2003) to create a cued-delay within-session TD procedure using solid food rewards (food pellets) for mice that is more similar to the procedure used for rats (Mar and Robbins, 2007).

Using a cued-delay within-session TD procedure we evaluated whether exposure to CUS alters executive function in GDNF-deficient mice (GDNF HET) and their WT littermate controls. Analyses indicated a reliable effect of stress on TD (indexed by %LL choices and %AUC) in GDNF HET but not WT mice, suggesting that impulsivity increased Post-Stress in GDNF HET but not in WT mice. Analysis of neuronal activation (cFos+ cell counts) in the OFC, PrL, NA core, and NA shell during TD in the Post-Stress condition, revealed a significant decrease in activation in OFC, NA core and NA shell, but not in PrL, suggesting that the decrease in OFC and NA activation in GDNF HET mice relative to controls is specific to the TD task, rather than being a non-specific, brain-wide effect. Interestingly, in our study, Post-Stress %AUC positively correlated with accumbens activity, but not with orbitofrontal activity. As impulsivity is indexed by a reduced %AUC, in our study impulsivity was negatively correlated with activity in these brain regions.

The prefrontal and orbitofrontal cortices and their interconnections with the NA, hippocampus and amygdala are central to decision making (Burton et al., 2015; Orsini et al., 2015). Value computation is thought to be related to the ventromedial prefrontal/OFC and the ventral striatum (van Duuren et al., 2008; Galtress and Kirkpatrick, 2010). OFC is thought to provide predictions about specific outcomes associated with stimuli, choices, and actions, especially their moment-to-moment value based on current internal states (Rudebeck and Murray, 2014). OFC lesions affect how long rats wait for rewards and shift the indifference point to the left in an adjusting delay paradigm (Mobini et al., 2002; Rudebeck et al., 2006); temporary OFC inactivation also increases impulsive choice in a TD paradigm when the delay is cued (similar to our study; Zeeb et al., 2010). The NA core is important in discounting delayed rewards as demonstrated by lesion studies (Galtress and Kirkpatrick, 2010; Valencia-Torres et al., 2012), pharmacological inactivation (Feja and Koch, 2015), or neuronal activation studies (da Costa Araujo et al., 2010). cFos immunoreactivity is a useful marker for the identification of brain regions of interest activated in TD tasks (da Costa Araujo et al., 2010); our analyses revealed a significant reduction in neural activation of both OFC, NA core and NA shell in stressed GDNF-deficient mice compared to controls. Since decision making circuits are modulated by DA (Assadi et al., 2009; Simon et al., 2011; Kayser et al., 2012; Saddoris et al., 2015) and NA-derived GDNF is an important retrograde enhancer of dopaminergic tone in the mesocorticolimbic system (Wang et al., 2010), abnormal regulation/maintenance of dopaminergic tone in stressed GDNF-deficient mice may underlie both their observed deficits in neuronal activation and their executive dysfunction. This possibility is supported by our observations that neuronal activation in NA core and NA shell was positively correlated with %AUC (negatively correlated with impulsivity).

Stress initiates intricate organismal responses that affect diverse cognitive and affective domains, in order to maximize adaptation to environmental challenges (Hermans et al., 2014). Acute and chronic stress generally have distinct effects: acute stress responses enable rapid detection of threat (reallocation of resources to a network promoting fear and vigilance, at the cost of the executive network), adequate responses and restoration of homeostasis when threats are no longer present (including normalization of emotional and cognitive processes; Hermans et al., 2014); chronic stress has adverse effects on physiology and behavior, with neuroendocrine modulators inducing long lasting structural changes in executive brain areas (Cook and Wellman, 2004; Dias-Ferreira et al., 2009; Anderson et al., 2016).

Stress alters dopaminergic tone and DA processing in key areas of the brain (Ahmad et al., 2010; Wanat et al., 2013; Belujon and Grace, 2015) and stress-level catecholamines (including norepinephrine) impair executive function and working memory (Thierry et al., 1976; Arnsten, 2011). In humans, acute psychosocial stress (Kimura et al., 2013), but not the threat of shock (Robinson et al., 2015), increases rates of delay discounting. Acute restraint stress induces alterations in effort-based discounting but not TD in rats (Shafiei et al., 2012). Chronic stress in humans (Fields et al., 2014) or chronic corticosterone exposure in rats (Torregrossa et al., 2012) increase impulsive choice.

Chronic stress has negative repercussions on vulnerable individuals, precipitating psychosis (Aiello et al., 2012; Holtzman et al., 2012, 2013), anxiety-related and mood disorders (Bale, 2006; Deppermann et al., 2014) or substance abuse (Sinha, 2008; Lijffijt et al., 2014). Neurotrophic factors such as GDNF and brain-derived neurotrophic factor (BDNF) are known to affect individual vulnerability to stress (Uchida et al., 2011; Bian et al., 2012; Bennett and Lagopoulos, 2014). Recent studies have shown that vulnerability to stress may be linked to epigenetic changes in GDNF expression in the ventral and/or dorsal striatum (Uchida et al., 2011) and thus may vary significantly between individuals. GDNF HET mice, having a single functional allele of the GDNF gene, have reduced levels of GDNF (Griffin et al., 2006) and may not be able to upregulate GDNF expression in the NA in response to stress, which may explain their increased vulnerability to stress manifested in alterations in their executive functions, as shown in the present study.

Our results indicating executive function impairments in response to stress are particularly relevant to substance abuse research, since maladaptive reactivity to stress is linked to addiction (Decressac et al., 2011; Miller, 2011; Ortiz-Ortiz et al., 2011). GDNF has recently been identified as an ethanol-responsive gene in the ventral tegmental area (VTA; Ahmadiantehrani et al., 2014) and is a known negative regulator of drug and alcohol addiction (Ron and Janak, 2005; Barak et al., 2015). Epigenetic alterations in GDNF expression could underlie both reactivity to stress and vulnerability to substance abuse, leading to health-risk behaviors.

Executive function impairments demonstrated in GDNF-deficient mice are also relevant to Parkinson’s Disease (PD), since GDNF-deficient mice are considered an animal model for aging-related Parkinsonism (Boger et al., 2010), exhibiting accelerated aging-related decline of DA and motor function (Griffin et al., 2006). Although PD is primarily considered a motor disorder, cognitive impairments are frequent in PD (Williams-Gray et al., 2006; Goldman and Weintraub, 2015): 20–38% of PD patients show mild cognitive impairment within 5 years of diagnosis (Caviness et al., 2007; Aarsland et al., 2009) and 70–90% of patients develop a frank dementia with disease progression (Gratwicke et al., 2015). Early PD patients show impairments in planning, working memory and inhibitory control, suggestive of frontal executive dysfunction (Lees and Smith, 1983; Taylor et al., 1986; Dirnberger and Jahanshahi, 2013). Impulsivity is common in PD, multiple forms of impulsivity resulting from changes in brain structure (Nombela et al., 2014), and being exacerbated by DA-based treatments (Wolters et al., 2008; Lee and Jeon, 2014). In PD, abnormal decision making is thought to be due to dysfunctions at the outcome evaluation stage of the decision-making process (Ryterska et al., 2013).

One obvious concern regarding using GDNF-deficient mice in our studies is the possibility for motor impairment interfering with the TD testing. However, in our study, GDNF mice were about 8–10 months old at the end of testing. At this age, GDNF-deficient mice do not show signs of motor impairment, motor symptoms appearing after 12 months of age, as previously documented by Boger et al. (2006). Therefore, in our study, GDNF-deficient mice were equivalent to pre-symptomatic Parkinsonian patients, which do not show motor impairment. Our results identify stress-induced executive dysfunction in a pre-symptomatic model of aging-related Parkinsonism as a potential predictive marker. Further studies are required to investigate whether the results obtained in GDNF-deficient mice can be found in other models of PD and are relevant to pre-symptomatic human carriers of PD-related gene mutations.

## Author Contributions

Experimental design: MB, CVB. Breeding and genotyping: MB. Temporal discounting: CVB, KO, BZY. Chronic unpredictable stress: MB, KO, BZY. Immunostaining and imaging: MB. Data analysis: MB, CVB. Wrote article: MB, CVB.

## Funding

This work was supported by National Institutes of Health grant NS090283 to MB.

## Conflict of Interest Statement

The authors declare that the research was conducted in the absence of any commercial or financial relationships that could be construed as a potential conflict of interest.
